# Information Disclosure Contents of the COVID-19 Data Dashboard Websites for South Korea, China, and Japan: A Comparative Study

**DOI:** 10.3390/healthcare9111487

**Published:** 2021-11-01

**Authors:** Bo Zhao, Mahyeon Kim, Eun Woo Nam

**Affiliations:** 1 Department of Health Administration, Graduate School, Yonsei University, 1 Yonseidae-gil, Gangwon-do, Wonju 26493, Korea; zhaobo@yonsei.ac.kr (B.Z.); kmh0208@yonsei.ac.kr (M.K.); 2 Healthy City Research Center, Institute of Health and Welfare, Yonsei University, 1 Yonseidae-gil, Gangwon-do, Wonju 26493, Korea

**Keywords:** COVID-19, information disclosure, dashboard website, South Korea, China, Japan

## Abstract

Official responses to the COVID-19 pandemic have prioritized information disclosure. Timely and comprehensive information released by the authorities is conveyed mainly through dashboards, which can better inform the public and help them prepare for the pandemic. However, there is limited evidence regarding the COVID-19 dashboard data presentation for South Korea, China, and Japan. This study aimed to describe the current COVID-19 situation in the three countries and compare the information disclosure content on their COVID-19 dashboards. Based on the COVID-19 data released and updated by each country’s official authorities, two dashboard websites used by many people in each country were selected. We conducted content analysis and developed a checklist (39 items in five categories: cases, testing, vaccines, health information, and additional items) based on the structure of each country’s COVID-19 dashboard website to assess COVID-19 information disclosure. Japan experienced the worst outbreak among the three countries. They all provided basic dynamic data displayed on the dashboard, while the performance in key categories varied substantially between the countries (South Korea: 30/39 items; China: 25/39 items; Japan: 30/39 items). Moreover, as part of the publicly accessible information recorded by each nation, there were differences in the key indicators published and important facts disclosed. Improvement in reporting techniques and disclosure methods will help countries communicate more effectively with the public and conduct more efficient public health research.

## 1. Introduction

In December 2019, a new virus that caused severe acute respiratory syndrome (SARS)—that is, the coronavirus disease of 2019 (COVID-19)—was reported in Wuhan, Hubei Province, China. It was initially called novel Coronavirus 2019-nCoV and later renamed SARS coronavirus 2 (SARS-CoV-2). It rapidly spread to other parts of China and other countries throughout the world, despite China’s massive efforts to contain the disease within Hubei. By 1 September 2021, the World Health Organization reported more than 217 million confirmed cases and 4.51 million deaths [1], implying an exponential growth in infections and deaths worldwide.

Researchers have addressed the priority of prompt data and information disclosure for disease preparedness and response that allows for a coordinated response to the COVID-19 pandemic [2]. Given the critical lessons gained from the worldwide response to Ebola, reliable data-sharing platforms are crucial for the timely production and dissemination of related knowledge [3]. Currently, there are various computer-based approaches that present COVID-19 data through different types of charts and figures by country using a comprehensive dashboard website, which is very useful for recognizing its situation and trend [4]. The dashboard was born as a communication tool based on the history of executive information systems from the 1980s [5]. Technological advances in the 1990s, including data warehousing and the development of thought leadership with the increased demand for evidence-based decision-making, pushed all managers to find tools that could more easily monitor performance. This chain of changes has led to the development of intelligence tools, including dashboard software [6]. Stephen Few [6], an information design thought leader, provided a working description of a dashboard, which is “a visual representation of the most critical information required to fulfill one or more objectives, condensed on a single screen so that it can be monitored and understood at a glance”. Dashboards primarily support quantitative measures of outputs and/or outcomes with some type of comparison. Dashboards are used to monitor critical information needed to accomplish an objective or a set of objectives. Quantitative information is generally best for this purpose [7,8]. Given the pressure to demonstrate compliance and evaluate the work of regulatory agencies and the public, social/nonprofit organizations have subsequently been influenced by the adoption of dashboards [9]. Furthermore, the use of dashboards as tools for monitoring and taking action to improve performance was pioneered by US state and local governments [10].

Similar to the original SARS-CoV epidemic of 2002/2003 [11] and seasonal influenza [12,13], the dashboard website as well as information systems and methods—including online real- or near-real-time mapping of disease cases and social media reactions to the disease spread, predictive risk mapping using population travel data, and tracing and mapping of super-spreader trajectories and contacts across space and time [14]—are indispensable for timely and effective pandemic monitoring and response [15]. Furthermore, with the production and rollout of COVID-19 vaccines, additional data and information are updated in related dashboards, which could provide and share information on the latest situation to the public. However, not all of the information that is used to accomplish monitoring goals can be expressed numerically. Simple lists are fairly common on dashboards, such as the inclusion of numbers, tables, and line charts. Under the effect of big data—which refers to the increased volume of data available, speed of data creation, and variety of digital data sources available for analytics [16]—adequate responses to and visual representation of extremely fast changes in COVID-19 status cannot be ensured in every country.

At the beginning of the COVID-19 crisis, South Korea and Japan seemed to be two of the countries first affected by the virus. Their proximity to China, the source of the outbreak, combined with their densely populated cities and a large elderly population, likely made these two countries face the highest risk [17]. The first confirmed case of COVID-19 in Japan and Korea was respectively reported on 15 January 2020 and 20 January 2020 [18]. South Korea [19], China [20], and Japan [21] could halt coronavirus transmission better than any other wealthy country during the pandemic’s early months. However, a new outbreak wave appeared to be emerging due to the Delta variant [22,23,24]. As of 13 October 2021, the total number of confirmed cases and mortality per 100,000 people in the three countries were, respectively, 336,742 and 5.04 in South Korea and 108,806 and 0.35 in China, with Japan having the highest number (1.71 million and 14.25) [25]. Rapid, effective, and efficient communication of epidemic information by governments has increased considerably following the emergence of SARS in 2003 and the avian influenza A (H7N9) epidemic in 2013, especially in China [26,27]. Since the World Health Organization declared COVID-19 to be a worldwide public health emergency, it is necessary to inform individuals about the health risks they and their communities face and provide them with accurate information updates [28]. The information provided by the government is critical for enabling the general public and researchers to overcome knowledge gaps and react quickly to catastrophes [29]. Given that the data dashboards have the strategic purpose of supporting the government’s management of the crisis, the analytical purpose of decision-making, and the operational purpose of monitoring and response [30], how do the three countries of South Korea, China, and Japan differ in their dashboard data presentation, reflecting the situation of their governments’ responses?

To address this issue, this study aimed to review and analyze the COVID-19-related information disclosure content provided by the dashboards of the South Korean, Japanese, and Chinese governments to track the coronavirus pandemic and its associated data as the situation unfolded globally. Some of these dashboards received data updates in near-real time (until 1 September 2021) with the official cooperation of the government. We compared the content of information disclosure items of these different country dashboards to discuss additional comprehensive policies and responses to support the fight against infectious disease outbreaks and the pandemic.

## 2. Methods

### 2.1. Study Design

In the process of data transmission, it is necessary to use clear and appropriate tools and methods that help the researchers visualize and present their understanding of the data—an approach through which the researchers could continue improve the quality of the created visualization [31]. Dashboards are a visual representation of data and are useful in theory [32]. A well-designed dashboard adds value by transforming data repositories into readable information and by supporting the visual identification of trends, patterns, and anomalies [33]. Thus, a set of three guidelines for establishing priorities was developed to assist in designing and implementing an effective dashboard initiative [34]. These guidelines are proper metrics, executive support, and simplicity. Metrics were given the priority to aid in the process of visual creation.

Metrics are frequently employed in a variety of fields and have a wide range of applications [35]. An initial focus on the objectives enables the definition of the proper metrics—effective metrics measure results in terms of defining actions and improvement rather than merely monitoring performance. Metrics may also be used to assess the efficacy of the visualization, which can be used to identify the optimal visualization. Furthermore, users can use comparison metrics to assess different features of the visual representation [31]. Given the importance of selecting meaningful and key metrics, first, a comparison of COVID-19 official dashboard websites and content analysis were conducted in several countries [36]. Based on the GQM (Goal-Question-Measurement) model, we described the questions we wanted to answer and which data we needed to collect to coherently answer them coherent, given the measurement aim [37,38]. Then we integrated the structure of each country’s COVID-19 dashboard website and developed a checklist (5 categories with a total of 39 items), which could be used as a rapid qualitative assessment tool, to assess COVID-19 information disclosure, as follows:

1. Cases (14 items);
2. Polymerase chain reaction testing (6 items);
3. Vaccines (8 items);
4. Healthcare information (4 items);
5. Additional items (7 items).

### 2.2. Subject Sources

**Figure 1:**
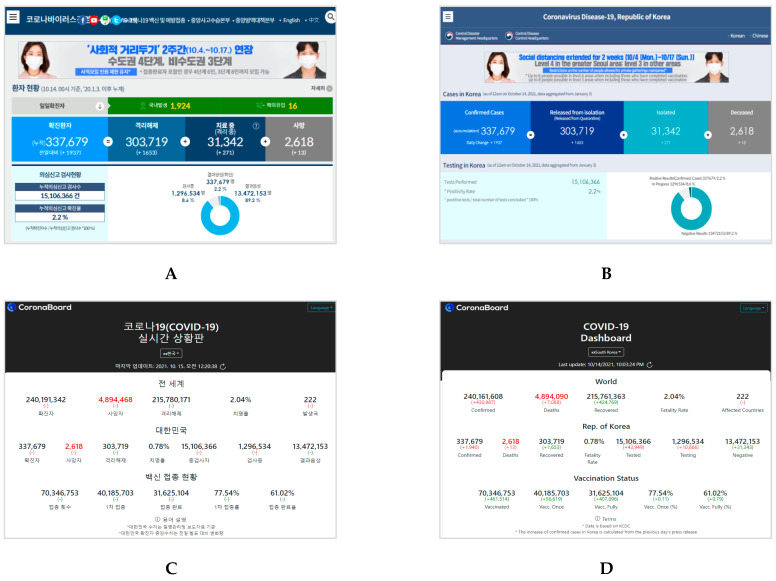
Homepages of the two dashboards in Korean (left) and English (right) for South Korea: (**A**) Coronavirus Disease-19, Republic of Korea. Center Disaster Management Headquarters. Center Disease Control Headquarters. URL: http://ncov.mohw.go.kr/ (In Korean, accessed on 14 October 2021) [40]; (**B**) Coronavirus Disease-19, Republic of Korea. Center Disaster Management Headquarters. Center Disease Control Headquarters. URL: http://ncov.mohw.go.kr/en/ (In English, accessed on 14 October 2021); (**C**) COVID-19 Dashboard in South Korea. Korea Disease Control and Prevention Agency. URL: https://coronaboard.kr/ (In Korean, accessed on 15 October 2021) [41]; (**D**) COVID-19 Dashboard in South Korea. Korea Disease Control and Prevention Agency. URL: https://coronaboard.kr/en/ (In English, accessed on 14 October 2021).

**Figure 2:**
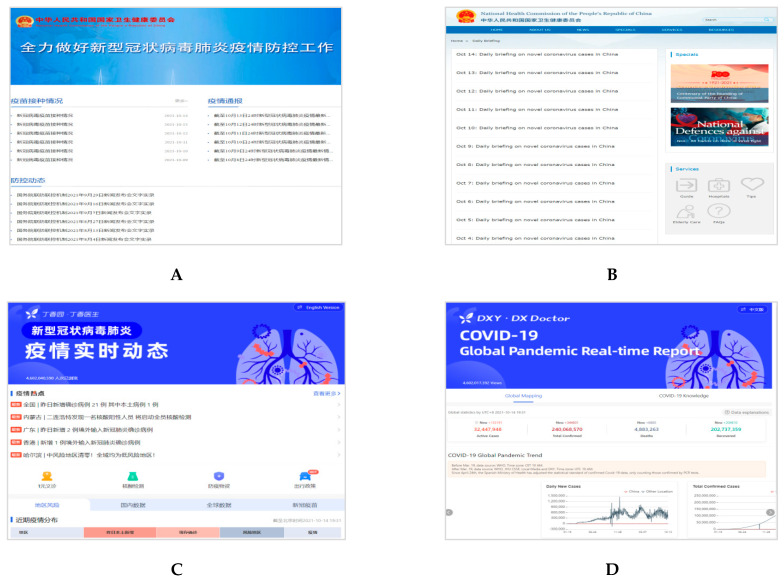
Homepages of the two dashboards in Chinese (left) and English (right) for China. (**A**) COVID-19 Prevention and Control. National Health Commission of the People’s Republic of China. URL: http://www.nhc.gov.cn/xcs/yqfkdt/gzbd_index.shtml (In Chinese, accessed on 14 October 2021) [42]; (**B**) Daily Briefing on Novel Coronavirus Cases in China. National Health Commission of the People’s Republic of China. URL: http://en.nhc.gov.cn/DailyBriefing.html (In English, accessed on 14 October 2021); (**C**) COVID-19 Global Pandemic Real-time Report. DXY, DX Doctor. URL: https://ncov.dxy.cn/ncovh5/view/pneumonia?from=dxy&source=&link=&share= (In Chinese, accessed on 14 October 2021) [43]; (**D**) COVID-19 Global Pandemic Real-time Report. DXY, DX Doctor. URL: https://ncov.dxy.cn/ncovh5/view/en_pneumonia?from=dxy&source=&link=&share= (In English, accessed on 14 October 2021).

**Figure 3:**
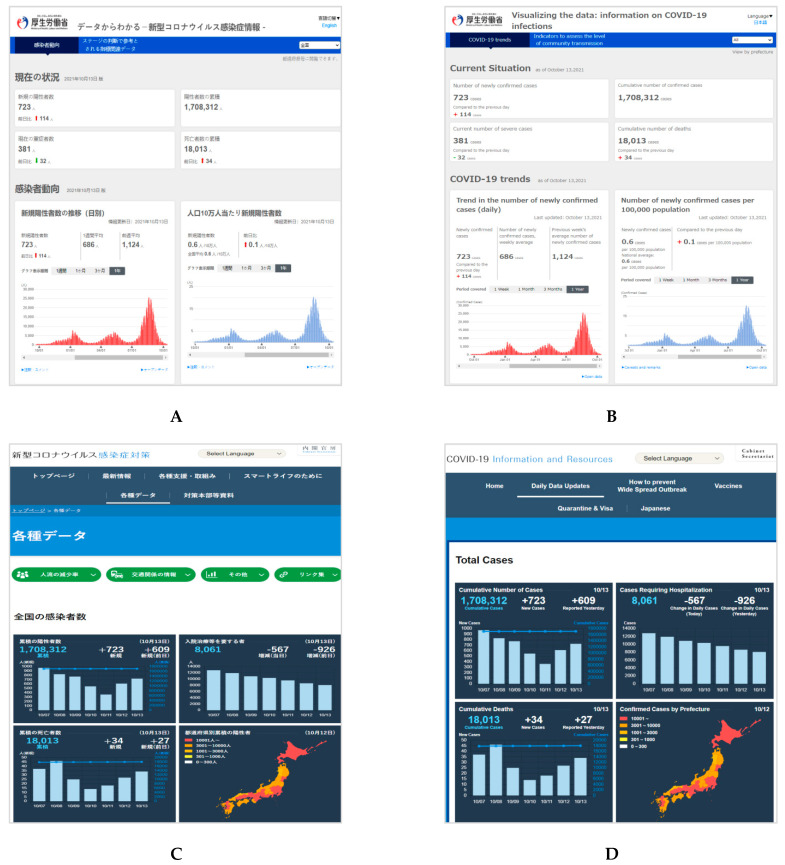
Homepages of the two dashboards in Japanese (left) and English (right) for Japan. (**A**) COVID-19 trends. Japan Ministry of Health Labor and Welfare. URL: https://covid19.mhlw.go.jp/extensions/public/index.html (In Japanese, accessed on 14 October 2021) [44]; (**B**) COVID-19 trends. Japan Ministry of Health Labor and Welfare. URL: https://covid19.mhlw.go.jp/extensions/public/en/index.html (In English, accessed on 14 October 2021); (**C**) COVID-19 Information and Resources. Office for Novel Coronavirus Disease Control, Cabinet Secretariat, Government of Japan. URL: https://corona.go.jp/dashboard/ (In Japanese, accessed on 14 October 2021) [45]; (**D**) COVID-19 Information and Resources. Office for Novel Coronavirus Disease Control, Cabinet Secretariat, Government of Japan. URL: https://corona.go.jp/en/dashboard/ (In English, accessed on 14 October 2021).

Based on the COVID-19 data released and updated by each country’s center for disease and prevention, we selected two official dashboard websites that were used by many people in each country (Figure 1, Figure 2 and Figure 3). All websites were captured in both the respective native language and English language of the interface (South Korea: Korean; China: Chinese; and Japan: Japanese). Considering that there may be information lost in translation between a native language interface and the English interface [39], we also added a URL link to the English interface and a reference to the native language under each captured website image.

### 2.3. Measures and Analysis

First, content analysis was conducted following the 5-category checklist and referring to the content of COVID-19 official dashboard websites in Korea, China, and Japan. Whether the information enumeration of the dashboard websites in the content items was involved in each category was contrasted, 39 items in total (Examples in Supplementary File S1).

If each country’s dashboard websites displayed the listed content, “yes” was marked in tables, otherwise, the content was marked “no”. The overall performance of each country in each category was aggregated and compared using radar maps. In addition, more detailed figures were added to display the provided specific information disclosure.

This study aimed to describe the current COVID-19 situation in the three countries and compare the information disclosure content on their dashboards.

## 3. Results

### Content of the COVID-19 Dashboards

Table 1, Table 2, Table 3, Table 4 and Table 5 show the results of comparing the three countries’ main dashboard information disclosure items. The performance of key categories and indicators varied between the countries (South Korea: 30/39 items; China: 25/39 items; and Japan: 30/39 items).

**Figure 4:**
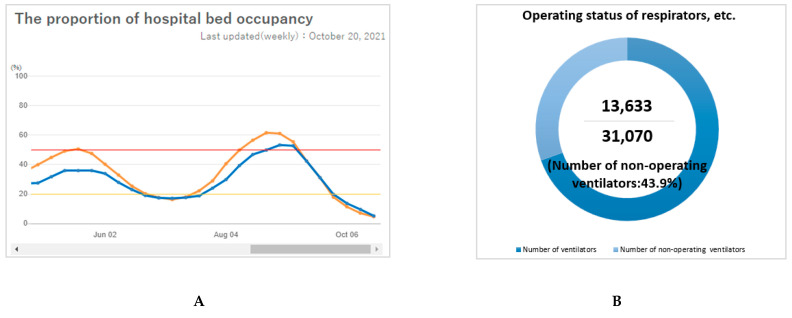
Healthcare information provided on the Japanese COVID-19 dashboards. (**A**): The proportion of hospital bed occupancy. (Note: Orange line: The proportion of the hospital bed occupancy for COVID-19, defined as the number of hospital beds used by COVID-19 cases divided by total hospital beds for COVID-19; Blue line: the proportion of the hospital bed occupancy for severe COVID-19, defined as the number of beds used by severe COVID-19 cases divided by total hospital beds for severe COVID-19) Source: Information on COVID-19 Infections. Japan Ministry of Health Labor and Welfare. https://covid19.mhlw.go.jp/extensions/public/en/index2.html (accessed on 30 October 2021) [44]; (**B**): Operational status of ventilators. Source: COVID-19 Information and Resources. Cabinet Secretariat, Government of Japan. https://corona.go.jp/dashboard/ (accessed on 14 October 2021) [45].

As for cases in Table 1, testing in Table 2, and vaccines in Table 3, the three countries displayed similar basic dynamic data on the dashboard. South Korea and Japan provided better information disclosure by category, such as gender and age, which was not provided on the Chinese dashboards. Concerning the health information in Table 4, more detailed information was provided on the Japanese COVID-19 dashboards. Health information such as numbers of present beds was provided on the Korean and Japanese dashboards. However, bed utilization rate and operational rate of ventilators were only clearly displayed on the Japanese dashboard (as Figure 4 shows), but this information was not found on the Korean and Chinese dashboards. However, information on COVID-19 registered hospitals was shown on all dashboards in South Korea and China. Regarding the seven additional items in Table 5, information on the status of the floating population was well presented on the Japanese dashboards. All three countries’ dashboards visually presented trend information, a risk level map, and a post refuting fake information or misinformation. Some misinformation about COVID-19-related policy (such as education, finance) and prevention were the top discussions [46]. Furthermore, a special item was found on the Chinese website—comment contact with the public—which provided an interactive channel of communication between the public and the National Health Commission.

**Figure 5:**
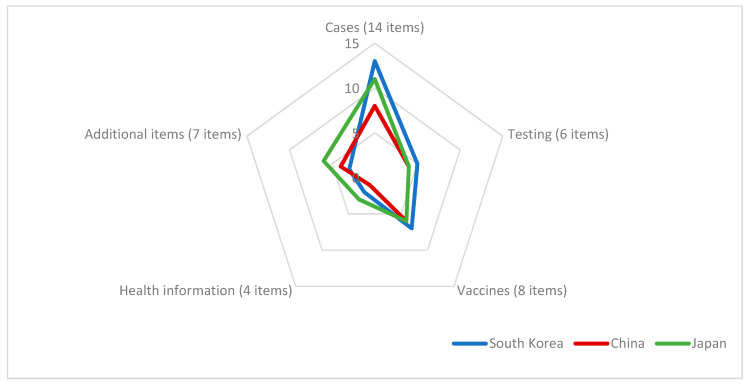
Performance of information disclosure on the COVID-19 dashboard in South Korea, China, and Japan.

Figure 5 presents an overview of the performance of information disclosure on the COVID-19 dashboard in South Korea, China, and Japan using a radar chart. To assess COVID-19 information disclosure, the checklist for the COVID-19 dashboard website’s structure consisted of 39 items in five categories (cases, testing, vaccines, health information, and additional items). Generally, South Korea did well in cases, testing, and vaccines, and Japan did well in both health information and additional items.

For the COVID-19 data, the crucial information was conveyed by presenting updated numbers and graphs, which also showed the underlying relationships between entities. In most information performance types, the principles of data presentation and graph illustration are similar, using different node sizes, colors, fonts, etc. to highlight the real-time data that are focused on the pandemic situation. To reduce the data-reading complexity, it would be better to provide more visual information as well as to enhance the readability of graphs, similar to what the Japanese dashboards do.

## 4. Discussion

This research provides an overview of three authorities’ disclosure performance in relation to COVID-19 pandemic information, based on findings in South Korea, China, and Japan as of September 2021. Compared with the date when China started its uncontrolled exponential growth (21 January 2020) (see Supplementary File S2), South Korea started 24 days later, and Japan started 42 days later [47]. The situation of COVID-19 in each country based on the cumulative and daily number of deaths normalized per 100,000 inhabitants presented similar growth trends as a function of a “shifted” time [48]. Although there seemed to be a weak association between the pandemic spread and the arrival date in various countries, this does not mean that the early lessons learned in affected countries would benefit all later affected countries, let alone that one country may be able to manage the different stages of the epidemic (such as Japan). After beating back the winter resurgence, COVID-19 resurged in South Korea and Japan. All three countries have already implemented COVID-19 vaccination, and South Korea and Japan launched their COVID-19 inoculation drives in February 2021, which were later than the start in China (December 2020). The Delta variant originally discovered in India in December has now become the most dominant and worrisome strain of the coronavirus, spreading globally. By July 2021, its presence was already confirmed by the World Health Organization to be present in 85 countries, including South Korea, China, and Japan, [49]. Concerns in each country have been growing as the Delta variant spreads. For this reason, the changing nature of the epidemic in different countries also reflects the need for governments and official departments to disclose available data and information to the public in an effective and timely manner [50].

We discovered that the three countries were proactive in disclosing important information about the COVID-19 pandemic by publishing COVID-19-themed content on their main dashboard websites. The most often provided information on these websites has been case updates, testing, vaccine surveillance, and advice on health information for the general public. The rapid and transparent reports and published data illustrate the power of providing important insights to guide complex policy decisions during the current pandemic [51].

The information disclosure released on COVID-19 dashboards was assessed during the pandemic surveillance in South Korea, China, and Japan. Epidemic surveillance summaries, as valuable sources of COVID-19 epidemiological data provided in the context of both entire populations and individuals, typically include crucial indicators, such as COVID-19 confirmed cases, mortality rates, and recoveries, which may all be used to monitor the epidemic trends [52,53]. Other studies have pointed out that epidemiological studies on COVID-19 rely heavily on confirmed case reports and related surveillance information [54,55]. In this study, there were differences in the major items published and important indicators disclosed as part of publicly accessible information by each country, as shown in the tables and figures. In summary, the performance and information disclosure on the two dashboards in Japan are not worse than those in Korea and China, and they provide detailed visual information using charts and figures. Given that Japan does not have a strong lockdown policy to weaken the spread of the infection [56], the situation is still not optimistic, despite the use of a good surveillance system [57,58].

The COVID-19 pandemic resulted in a huge infodemic, with the population being inundated with large amounts of information. In the early stages of the pandemic, misconceptions of the general public in the United States and the United Kingdom indicated the need for timely and effective information disclosure by public health authorities [59]. However, such disclosure is impossible if the information and data are disorganized. The dissemination of unbiased and highly accurate information could assist the public in self-protection and could result in a reduction in the pandemic [60]. Misinformation and disinformation would cause fear and increase the danger of an outbreak. Another finding based on the Chinese city websites underscored the need for well labeled content categories, appropriate arrangement, and a clear focus on the audience’s demands, while also revealing information during an emergency [29]. In addition, previous studies in the United States emphasize the importance of adhering to usability guidelines for information posted on these websites [61].

The public should be informed about the type of indicators used. Our findings indicate discrepancies between the key metrics provided in summary reports from the different nations studied in the current research. According to our research on indicators used in epidemic-monitoring summaries, there is still no unanimity on this subject among governments. Johns Hopkins University hosts one of the most frequently cited web-based interactive dashboards, and it is a good example of showing speed and the timely communication of data [62,63]. The information this dashboard provides has helped influence public health decision-making and worldwide communication. Hu et al.’s study also suggested substantial differences in message and data templates for producing epidemic surveillance summaries and verified case reports, which might have to be updated using consistent protocols and standards, based on the major implications for disclosure of information and risk communication throughout a pandemic or catastrophic incident [29].

Regarding the confirmed cases and tracking information, it is important to note that virtually all information supplied by the authorities may be enhanced for greater accuracy and completeness. Not simply data/numbers update or display, but also through easy to understand and accept graphical information that increases readability. Public health emergencies require quick information disclosure and effective data exchange to help all stakeholders make educated decisions [64]. Therefore, it is recommended for countries to learn from one another and develop uniform indicators and standards for pandemic-related messages that are presented in a clear and appropriate manner. Furthermore, vital information should be updated using standard procedures and indicators that the public can readily understand, so that researchers can properly evaluate it on a regular basis.

### Implications and Limitations

This study had several limitations. First, this study chose two dashboards in each country, and the checklist we developed as a rapid qualitative assessment tool was somewhat subjective. It is therefore likely that certain changes in information disclosure between countries may be ignored or overestimated. The comparison could be further improved by adding more scientific quantitative studies. Second, due to the rapidly changing COVID-19 situation in different countries, local information disclosure performance may change over time. Future studies might need to shed light on how the authorities deal with the pandemic situation. Nevertheless, official websites and data have been assumed to be the most authoritative and reliable information disclosure sources. Therefore, this research considered online dashboards released by these authorities as key public resources. Real-time roll-out of COVID-19 updates available through dashboard platforms may be useful for informing the public [61]. As a result, the findings of this study may provide evidence for reference when implementing future policymaking and data visualization updates.

## 5. Conclusions

This study assessed content released based on the pandemic surveillance summaries and related information in South Korea, China, and Japan. As part of the publicly accessible information recorded by each nation, there were differences in the key indicators published and important facts disclosed in the study. Based on a comparison of information and data disclosed during the COVID-19 outbreak among the three countries, South Korea and China can improve their disclosure by learning from the detailed and visual information displayed on Japan’s dashboards.

Encouragement of the dissemination of information related to emergency health circumstances is emphasized, as is providing the public with regular channels via whatever accurate, advanced information may be disseminated. Further improvements in reporting techniques and disclosing methods will help nations communicate more effectively and conduct more efficient public health research between countries.

## Figures and Tables

**Table 1:** Content comparison for “cases” on the COVID-19 dashboard in South Korea, China, and Japan.

Items		South Korea	China	Japan
Cases	1. Confirmed cases categorized by			
	Domestic/Overseas	yes	yes	yes
	2. Number of new confirmed cases (daily/weekly)	yes	yes	yes
	3. Total number of confirmed cases	yes	yes	yes
	4. Situation regarding region	yes	yes	yes
	5. Situation regarding cluster	yes	no	yes
	6. Status of confirmed cases by gender	yes	no	yes
	7. Status of confirmed cases by age	yes	no	yes
	8. Case inpatients	yes	no	yes
	9. Number of severe cases	yes	yes	yes
	10. Number of close contacts/suspected cases	yes	yes	no
	11. Number of no-symptom cases	no	yes	no
	12. Number of cured cases	yes	yes	yes
	13. Number of deaths	yes	yes	yes
	14. Route information on confirmed cases	yes	yes	no

**Table 2:** Content comparison of “testing” for the COVID-19 dashboard in South Korea, China, and Japan.

Items		South Korea	China	Japan
Testing	1. Tests performed	yes	yes	yes
	2. Tests concluded (daily/cumulative)	yes	yes	yes
	3. In progress	yes	no	yes
	4. Positive/negative results	yes	no	yes
	5. Positive rates	yes	yes	no
	6. Testing capacity	no	yes	no

**Table 3:** Content comparison of “vaccines” for the COVID-19 dashboard in South Korea, China, and Japan.

Items		South Korea	China	Japan
Vaccines	1. Vaccinations given total			
	First dose total/Second dose total	yes	yes	yes
	2. First dose conducted rate	yes	yes	yes
	3. Both doses/completion conducted rate	yes	yes	yes
	4. Status by vaccine type	yes	no	no
	5. Vaccination status by gender	no	no	yes
	6. Vaccination status by subjective	yes	yes	no
	7. Vaccination status by region	yes	yes	yes
	8. Vaccination information	yes	yes	yes

**Table 4:** Content comparison of “healthcare information” for the COVID-19 dashboard in South Korea, China, and Japan.

Items		South Korea	China	Japan
Healthcare information	1. Numbers of present beds (general/intensive care unit)	yes	no	yes
	2. Bed utilization rate	no	no	yes
	3. Operational status/rate of ventilators	no	no	yes
	4. COVID-19 registered hospital	yes	yes	no

**Table 5:** Content comparison of “additional items” for the COVID-19 dashboard in South Korea, China, and Japan.

Additional Items		South Korea	China	Japan
Status of human flow	1. Trend of flow of people at major subway stations/street/tourism sites across country (before and after)	no	no	yes
	2. Population changes (before and after)	no	no	yes
Status of telecommuting work	3. Status of telecommuting work information provided	no	no	yes
Visualization	4. Trend information (charts/arrowheads)	yes	yes	yes
	5. Risk level map (color)	yes	yes	yes
Misinformation response	6. Post of refuting the fake or misinformation	yes	yes	yes
Comment contact with the public	7. Comment contact/reply provided	no	yes	no

## Data Availability

Data available in a publicly accessible repository.

## Supplementary Materials

The following are available online at www.mdpi.com/article/10.3390/healthcare9111487/s1, Supplementary File S1: Examples of Content comparison on the COVID-19 dashboard, Supplementary File S2: Related study about the COVID-19 situations in Korea, China, and Japan.
