# Supplementary material for: Information Disclosure Contents of the COVID-19 Data Dashboard Websites for South Korea, China, and Japan: A Comparative Study
Source: Healthcare (Basel). 2021 Nov 1;9(11):1487. doi: 10.3390/healthcare9111487 (PMC8619658; doi:10.3390/healthcare9111487)
Supplement: Supplementary file 1 [file healthcare-09-01487-s001.zip › Supplementary 2(S2). Related study about the COVID-19 situations in Korea, China, and Japan.pdf]

S2. Related study about the COVID-19 situations in Korea, China, and Japan.

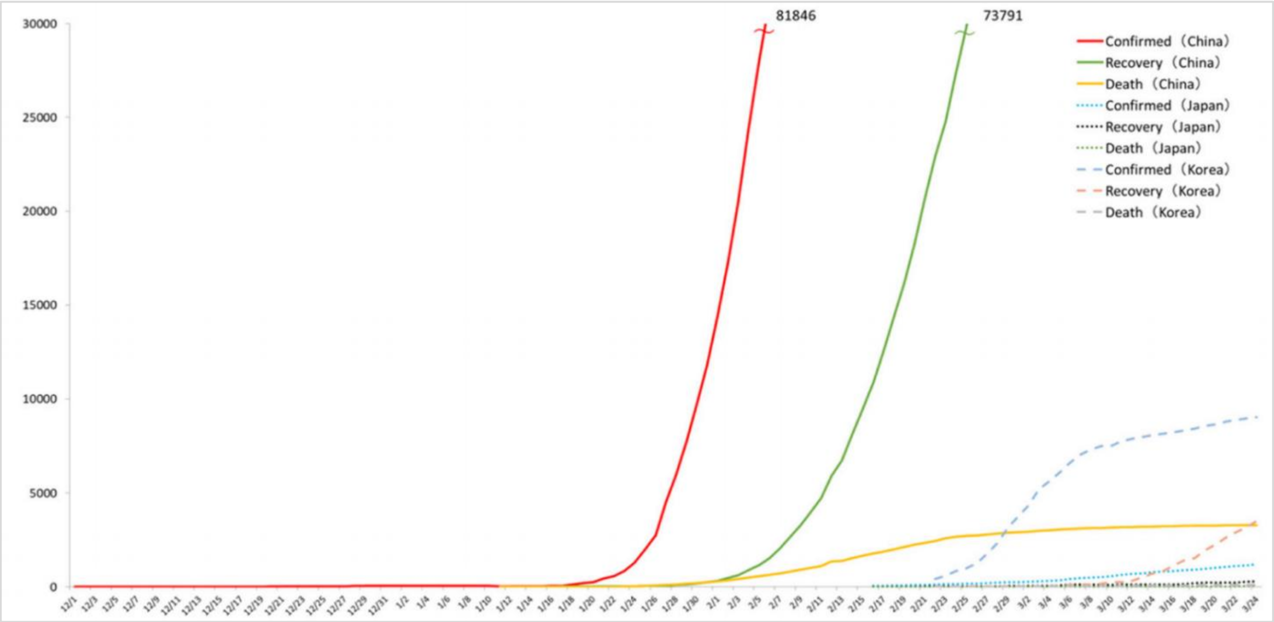

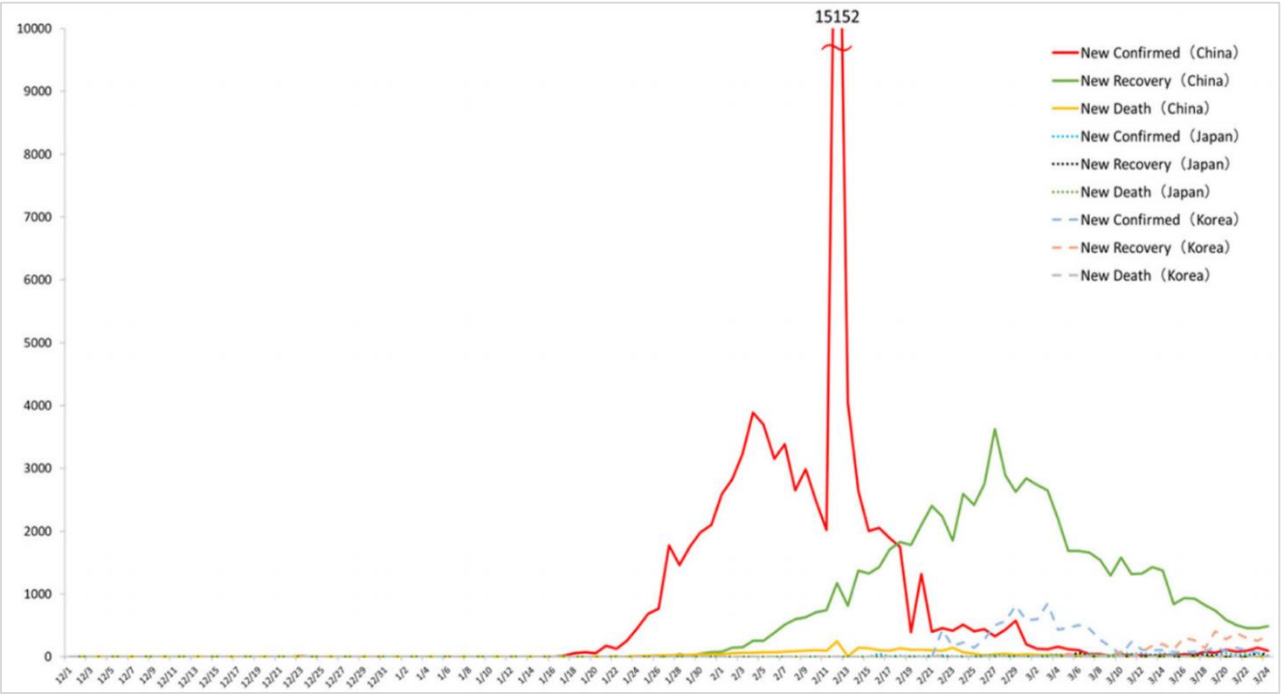

Figure S1. a. Total number of confirmed, recovered and death in China, Korea and Japan. Panel b. Daily increase of confirmed, recovered and death in China, Korea and Japan [Shaw, et al. 2020].

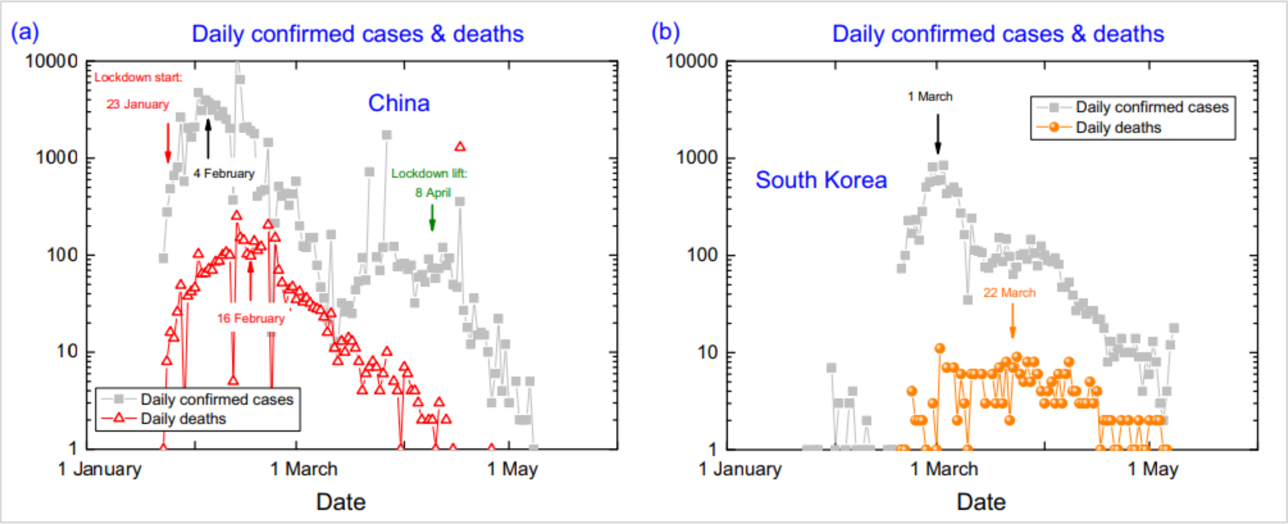

Figure S2. Time variation of daily confirmed cases and deaths in: (a) China, (b) South Korea [Knafo W. 2020].

[Knafo W. 2020] found that the variation of the daily death number follows that of the daily confirmed cases, with a delay of 5 10 days. In China, a significant decrease of daily confirmed cases was observed 10 days after the setup of lockdown, and the epidemic peak in the number of daily deaths was observed 10 days later. After this peak, the number of daily deaths has decreased within an exponential decay. Two months later, lockdown was lifted on 8 April. At this date, there were a few daily deaths and ≈ 50–100 daily confirmed cases in China. The case of South Korea is unique: after an early increase of daily confirmed cases, this number reached a maximum of ≈ 1000 before strongly decreasing. By the end of April, less than 2 daily deaths and ≈ 10 daily confirmed cases were reported. Before May, the number of daily deaths has always been contained to less than 10 in South Korea.

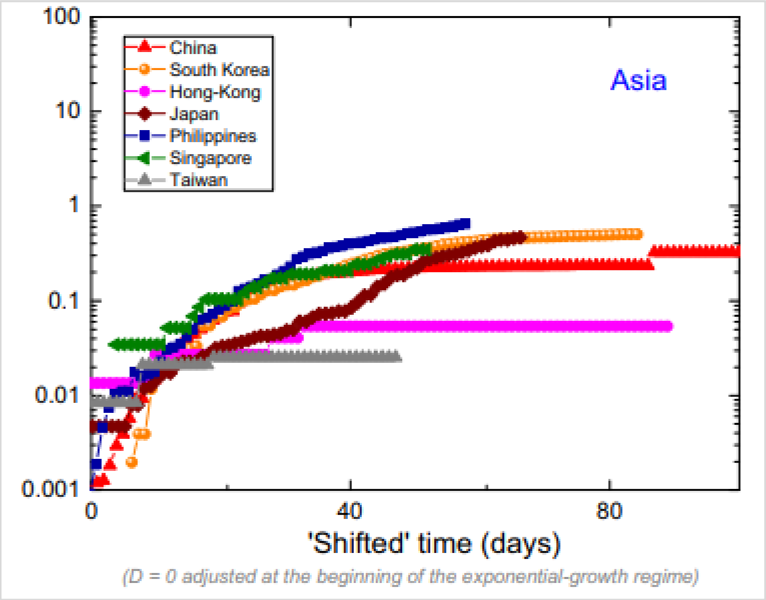

Figure S3. Cumulative and daily death tolls per 100,000 inhabitants in Asia. Cumulative deaths per 100,000 inhabitants versus "shifted" time in Asian countries [Knafo W. 2020].

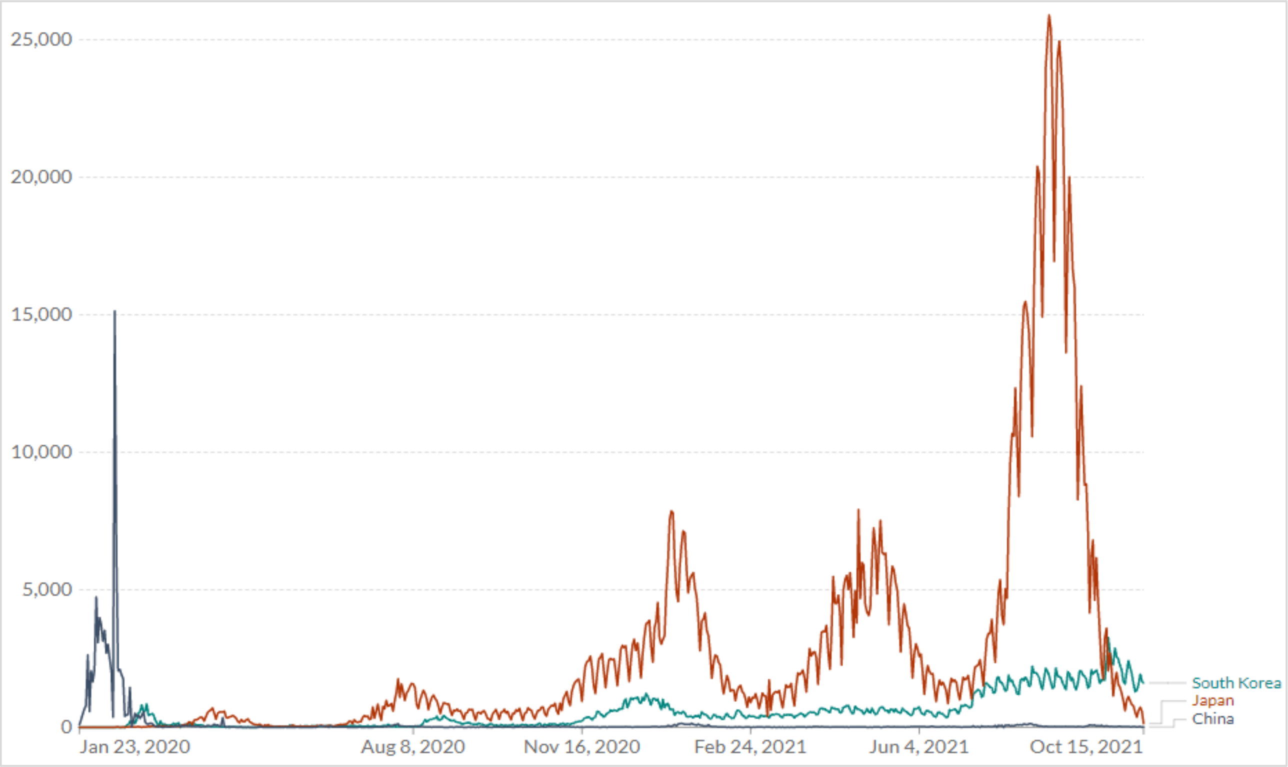

Figure S4. Daily new confirmed COVID-19 cases in South Korea, China, and Japan by Oct 15, 2021. Source: Our World in Data from John Hopkins University CSSE COVID-19 Data, https://ourworldindata.org/coronavirus.

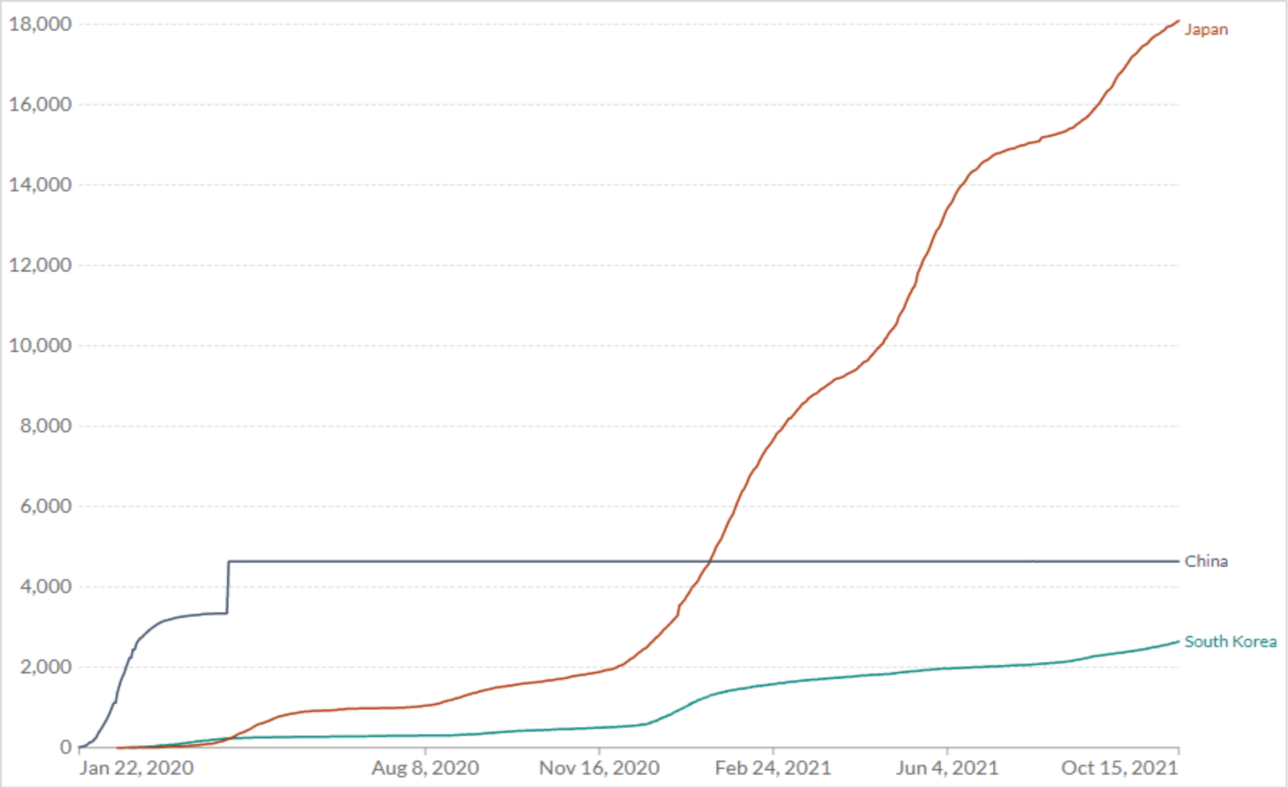

Figure S5. Cumulative confirmed COVID-19 deaths in South Korea, China, and Japan by Oct 15, 2021. Source: Our World in Data from John Hopkins University CSSE COVID-19 Data, https://ourworldindata.org/coronavirus.

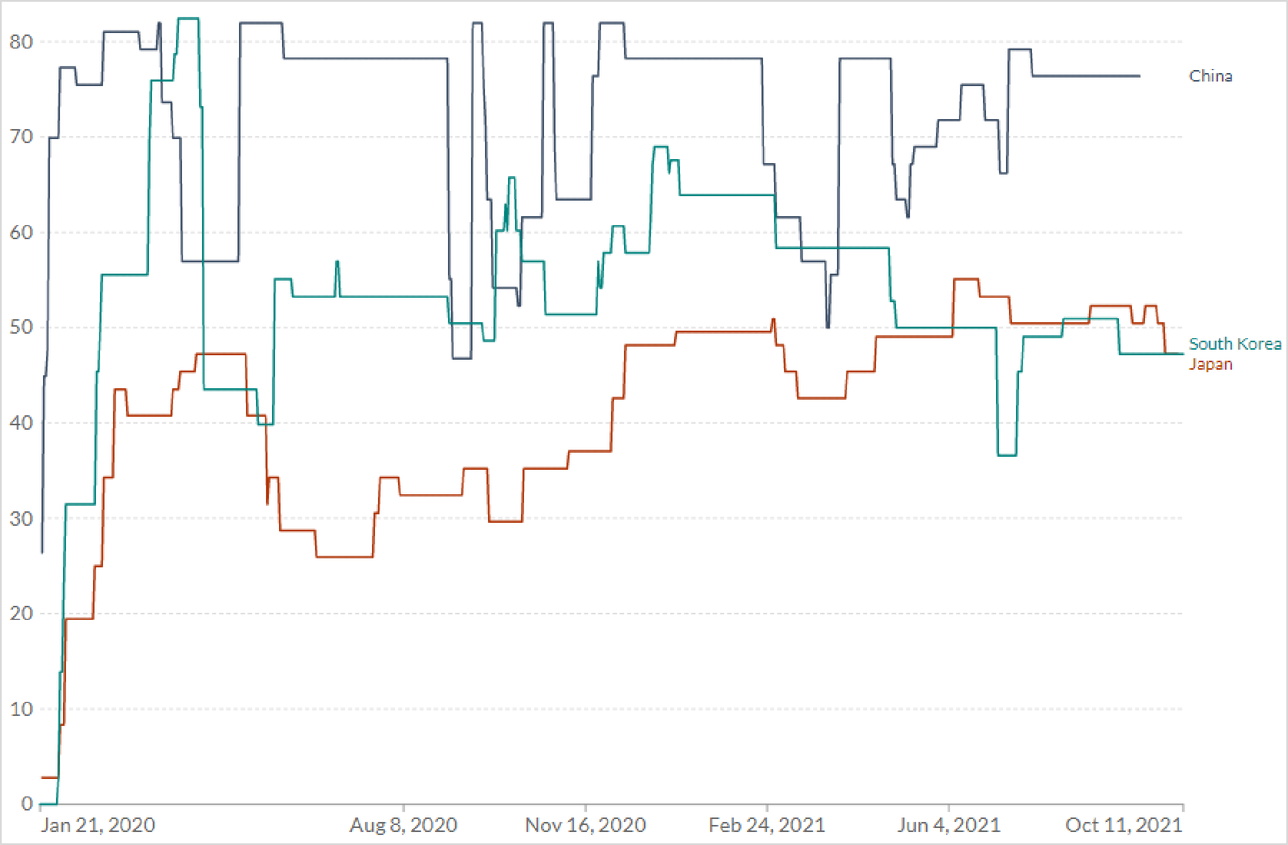

Figure S6. COVID-19 Stringency Index in South Korea, China, and Japan by Oct 15, 2021 (This is a composite measure based on nine response indicators including school closures, workplace closures, and travel bans, rescaled to a value from 0 to 100 (100 = strictest). If policies vary at the subnational level, the index is shown as the response level of the strictest sub-region.) Source: Our World in Data from John Hopkins University CSSE COVID-19 Data, https://ourworldindata.org/coronavirus.

References:

Shaw R, Kim YK, Hua J. Governance, technology and citizen behavior in pandemic: Lessons from COVID-19 in East Asia. Progress in disaster science. 2020 Apr 1; 6:100090.

Knafo W. COVID-19: Monitoring the propagation of the first waves of the pandemic. 4open. 2020, 3, 5.
