# Supplementary material for: Information Disclosure Contents of the COVID-19 Data Dashboard Websites for South Korea, China, and Japan: A Comparative Study
Source: Healthcare (Basel). 2021 Nov 1;9(11):1487. doi: 10.3390/healthcare9111487 (PMC8619658; doi:10.3390/healthcare9111487)
Supplement: Supplementary file 1 [file healthcare-09-01487-s001.zip › Supplementary 1(S1). Examples of Content comparison on the COVID-19 dashboard.pdf]

S1. Examples of Content comparison on the COVID-19 dashboard

Table S1. Examples of Content comparison for "cases" on the COVID-19 dashboard

**Items**		**Examples** (as of October 15, 2021)
Cases	1. Confirmed cases categorized by<br>Domestic<br>Overseas	On Oct 13, 2021, Chinese mainland reported 21 new cases of confirmed infections (20 imported cases) [1]
	2. Number of new confirmed cases (daily/weekly)	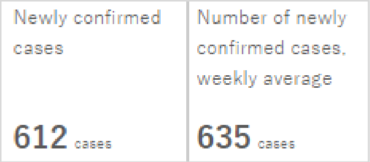 [2]
	3. Total number of confirmed cases	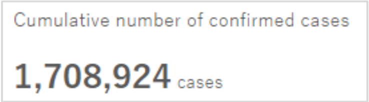 [2]
	4. Situation regarding region	On Oct 13, 2021, confirmed cases in Chinese mainland (6 in Yunnan province, 3 in Tianjin municipality, 2 in Shandong province, 2 in Guangdong province, 2 in Guangxi Zhuang autonomous region, 1 in Liaoning province, 1 in Shanghai municipality, 1 in Zhejiang province, 1 in Henan province and 1 in Hubei province, 2 confirmed cases converting from asymptomatic cases) [1]
	5. Situation regarding cluster	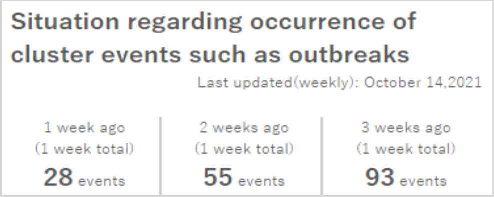 [2]
	6. Status of confirmed cases by gender	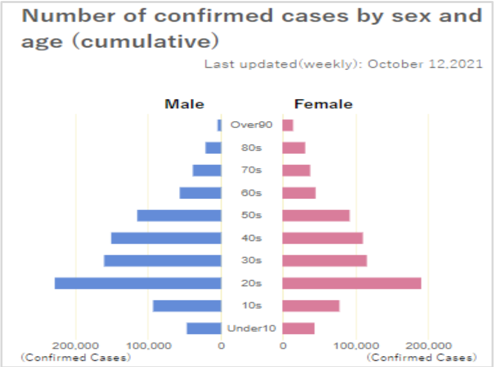 [2]
	7. Status of confirmed cases by age	
	8. Case inpatients	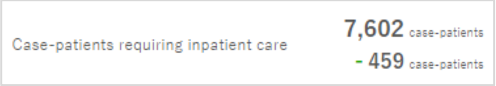 [2]
	9. Number of severe cases	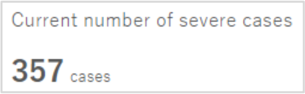 [2]
	10. Number of close contactee/suspected cases	1,208,390 people had been identified as having had close contact with infected patients. [1]
	11. Number of no-symptom cases	370 asymptomatic cases were still under medical observation (including 358 imported cases). [1]
	12. Number of cured cases	36 patients were released from hospital after being cured, and in all 91,212 patients had been cured and discharged from hospital. [1]

13. Number of deaths	Cumulative number of deaths 18,044 cases [2]
14. Route information of confirmed cases	The movement path of confirmed patients will be disclosed. [3]

[1]: National Health Commission of the People's Republic of China. Daily briefing on novel coronavirus cases in China. http://en.nhc.gov.cn/2021-10/14/c_84781.htm.
[2]: Japan Ministry of Health Labor and Welfare. Information on COVID-19 Infections. https://covid19.mhlw.go.jp/en/.
[3]: Korea Centers for Disease Control and Prevention. Coronavirus Disease-19, Republic of Korea. http://ncov.mohw.go.kr/bdBoardList_Real.do?brdId=1&brdGubun=12&ncvContSeq=&contSeq=&board_id=&gubun=.

Table S2. Examples of Content comparison of "testing" for the COVID-19 dashboard

**Items**		**Examples** (as of October 15, 2021)
Testing	1. Tests performed	Tests Performed 15,152,526 [1]
	2. Tests concluded (daily/cumulative)	Virus tests conducted Daily 1,045,158 Total 312,222,535 [2]
	3. In progress	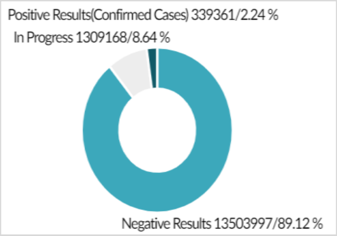
	4. Positive/negative results	[1]
	5. Positive rates	* Positivity Rate 2.2% * postitive tests / total number of tests concluded * 100% [1]
	6. Testing capacity	PCR testing capacity 906,719 [2]

[1]: Korea Centers for Disease Control and Prevention. Coronavirus Disease-19, Republic of Korea. http://ncov.mohw.go.kr/en/.
[2]: GOV.UK Coronavirus (COVID-19) in the UK. https://coronavirus.data.gov.uk/details/testing.

Table S3. Content comparison of "vaccines" for the COVID-19 dashboard

<table>
<tr><th>Items</th><th></th><th>Example (as of October 15, 2021)</th></tr>
<tr><td>Vaccines</td><td>1. Vaccinations given total</td><td>Vaccinations given
Total
94,560,905
[1]</td></tr>
<tr><td></td><td>2. First dose total
3. Second dose total</td><td>People vaccinated
First dose total 49,291,665
Second dose total 45,269,240
[1]</td></tr>
<tr><td></td><td>4. First dose conducted rate
5. Both doses/completion conducted rate</td><td>77.64% (+0.10) Vacc. Once (%)
61.90% (+0.87) Vacc. Fully (%)
[2]</td></tr>
<tr><td></td><td>6. Status by vaccine type</td><td>Korea Vaccination by Type
Total vaccinated: 61,688,119 (Astrazeneca: 21,864,945, Pfizer: 38,357,466, Janssen: 1,465,708
KCDC Data 2021-10-15 00:00
[2]</td></tr>
<tr><td></td><td>7. Vaccination status by gender</td><td><table>
<tr><th>Gender</th><th>Male</th><th>Female</th></tr>
<tr><td>Population</td><td>61,794,804</td><td>64,850,221</td></tr>
<tr><td>Number of first dose (rate)</td><td>41,801,261 (67.65%)</td><td>44,615,457(68.80%)</td></tr>
<tr><td>Number of second dose (rate)</td><td>36,523,412 (59.10%)</td><td>39,861,408 (61.47%)</td></tr>
</table>[3]</td></tr>
<tr><td></td><td>8. Vaccination status by subjective</td><td>Vaccination status by vaccination target in the second quarter (as of April 24 2021, unit: person)<table>
<tr><th>Division</th><th>Vaccination Subjects</th><th>Person who made content</th></tr>
<tr><td>Nursing hospital (≧ 65)</td><td>208,734</td><td>145,388</td></tr>
<tr><td>Disability population</td><td>355,388</td><td>233,921</td></tr>
<tr><td>Health care workers</td><td>272,043</td><td>142,537</td></tr>
<tr><td>People with chronic disease</td><td>77,969</td><td>18,537</td></tr>
</table>[4]</td></tr>
<tr><td></td><td>9. Vaccination status by region</td><td>Vaccinations by City/Province (Unit: number of persons)
First Dose Administered | Fully Vaccinated | Booster Dose Administered
[5]</td></tr>
<tr><td></td><td>10. Vaccination information</td><td>Q&A Fact Sheet › How does COVID-19 vaccination protect your body from the coronavirus?
Q&A Fact Sheet
[5]</td></tr>
</table>

[1]: GOV.UK Coronavirus (COVID-19) in the UK. https://coronavirus.data.gov.uk/details/vaccinations.
[2]: COVID-19 Dashboard in South Korea. https://coronaboard.kr/en/.
[3]: Government Chief Information Officers' Portal, Japan. https://cio.go.jp/c19vaccine_dashboard.
[4]: Korea Centers for Disease Control and Prevention. Coronavirus Disease-19, Republic of Korea. http://ncov.mohw.go.kr/.
[5]: Korea Centers for Disease Control and Prevention. COVID-19 Vaccination. https://ncv.kdca.go.kr/eng/.

Table S4. Examples of Content comparison of "healthcare information" for the COVID-19 dashboard

<table>
<tr><th>Items</th><th></th><th>Example (as of October 15, 2021)</th></tr>
<tr><td>Healthcare information</td><td>1. Numbers of present beds</td><td>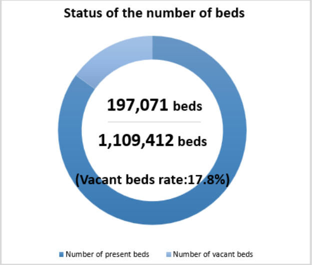

[1]</td></tr>
<tr><td></td><td>2. Bed utilization rate</td><td>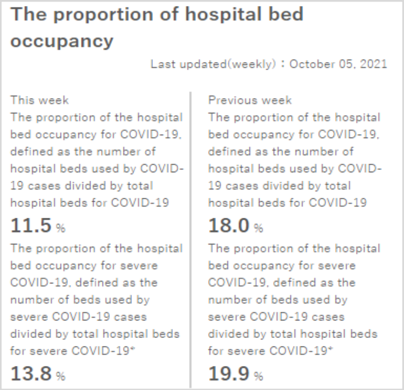

[2]</td></tr>
<tr><td></td><td>3. Operational status/ rate of ventilators</td><td>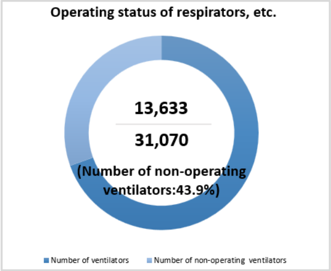

[1]</td></tr>
<tr><td></td><td>4. COVID-19 registered hospital</td><td>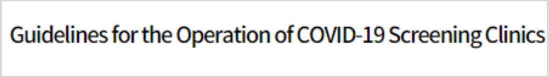

[3]</td></tr>
</table>

[1]: Office for Novel Coronavirus Disease Control, Cabinet Secretariat, Government of Japan. COVID-19 Information and Resources. https://corona.go.jp/dashboard/
[2]: Japan Ministry of Health Labor and Welfare. Information on COVID-19 Infections. https://covid19.mhlw.go.jp/extensions/public/en/index2.html.
[3]: Korea Centers for Disease Control and Prevention. Guidelines. http://ncov.mohw.go.kr/en/guidelineView.do?brdId=18&brdGubun=181&dataGubun=&ncvContSeq=2937&contSeq=2937&board_id=&gubun=.

Table S5. Examples of Content comparison of "additional items" for the COVID-19 dashboard

Additional Items		Example (as of October 15, 2021
Status of human flow	1. Trend of flow of people at major subway stations/street/tourism sites across country (before and after)	Changes in the flow of people at major stations and downtown areas nationwide<br>(rate of increase / decrease in the 15:00 range on Thursday, October 14, 3rd year of Reiwa) (comparison with the previous day (13th), before the spread of infection *)<br>Changes in the flow of people in major tourist destinations nationwide (rate of increase / decrease in the 15:00 range on Thursday, October 14, 3rd year of Reiwa)<br>Shibuya Center Street: ↓21.8% Comparison with the previous day; ↓53.1% Comparison with before the spread of infection<br>Harajuku Station: ↓10.2% Comparison with the previous day; ↓46.1% Comparison with before the spread of infection<br>[1]
	2. Population changes (before and after)	Analysis of population changes nationwide<br>(rate of increase / decrease in the 15:00 range on Thursday, October 14, 3rd year of Reiwa) (comparison with the previous day (13th), before the spread of infection *)<br>Inter-prefecture movement analysis during Golden Week in 2020<br>[1]
Status of telecommuting work	3. Status of telecommuting work information provided	Announcement status of implementation status of telework, etc. (see table below) [1]
Visualization	4. Trend information	(see figure below) [2]

Announcement status of implementation status of telework, etc.

Publication of the Implementation Status of Telework, etc. [2021.10.13]

	Number of responses	Number of listed companies	Percentage of listed companies	Total of listed companies
All	1,034	460	12.1%	3,800
Hokkaido	19	1	2.1%	48
Tohoku	18	2	4.3%	46
Hokuriku	8	3	3.0%	101
Metropolitan area	696	316	13.6%	2,325

[1]

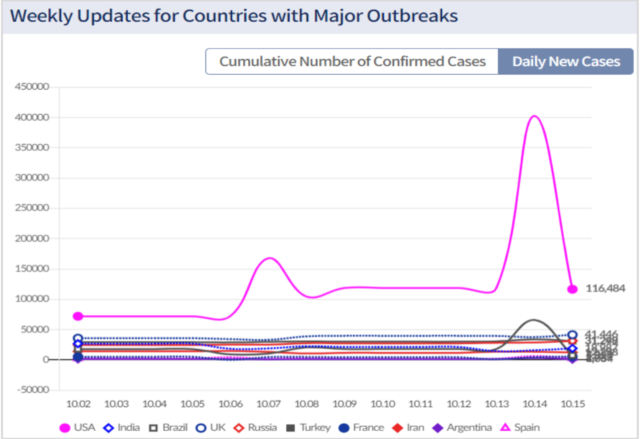

[2]

<table>
<tr><td></td><td>5. Risk level map (color)</td><td>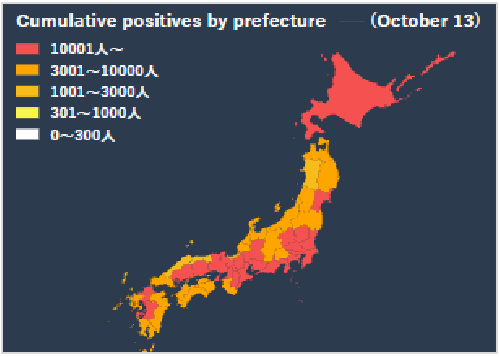 [1]</td></tr>
<tr><td>Misinformation response</td><td>6. Post of refuting the fake or misinformation</td><td>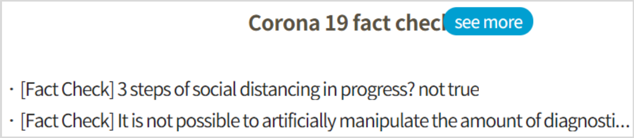 [2]</td></tr>
<tr><td>Comment contact with the public</td><td>7. Comment contact/reply provided</td><td>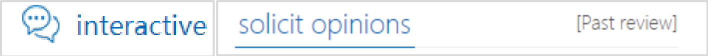 [3]</td></tr>
</table>

[1]: Office for Novel Coronavirus Disease Control, Cabinet Secretariat, Government of Japan. COVID-19 Information and Resources. https://corona.go.jp/dashboard/.
[2]: Korea Centers for Disease Control and Prevention. Coronavirus Disease-19, Republic of Korea. http://ncov.mohw.go.kr/.
[3]: National Health Commission of the People's Republic of China. http://www.nhc.gov.cn/.
